# Context matters: the psychoneurobiological determinants of placebo, nocebo and context-related effects in physiotherapy

**DOI:** 10.1186/s40945-020-00082-y

**Published:** 2020-06-11

**Authors:** Giacomo Rossettini, Eleonora Maria Camerone, Elisa Carlino, Fabrizio Benedetti, Marco Testa

**Affiliations:** 1 Department of Neuroscience, Rehabilitation, Ophtalmology, Genetics, Maternal and Child Health, University of Genova, Campus Universitario di Savona, via Magliotto 2, 17100 Savona, Italy; 2 Department of Neuroscience, University of Turin Medical School, Turin, Italy; 3 Plateau Rosà Laboratories, Plateau Rosà Laboratories, Zermatt, Switzerland

**Keywords:** Contextual factors, Placebo effect, Nocebo effect, Physical therapy modalities, Pain, Expectation, Conditioning, Rehabilitation, Therapeutic outcome, Learning

## Abstract

**Background:**

Placebo and nocebo effects embody psychoneurobiological phenomena where behavioural, neurophysiological, perceptive and cognitive changes occur during the therapeutic encounter in the healthcare context. Placebo effects are produced by a positive healthcare context; while nocebo effects are consequences of negative healthcare context. Historically, placebo, nocebo and context-related effects were considered as confounding elements for clinicians and researchers. In the last two decades this attitude started to change, and the understanding of the value of these effects has increased. Despite the growing interest, the knowledge and the awareness of using the healthcare context to trigger placebo and nocebo effects is currently limited and heterogeneous among physiotherapists, reducing their translational value in the physiotherapy field.

**Objectives:**

To introduce the placebo, nocebo and context-related effects by: (1) presenting their psychological models; (2) describing their neurophysiological mechanisms; (3) underlining their impact for the physiotherapy profession; and (4) tracing lines for future researches.

**Conclusion:**

Several psychological mechanisms are involved in placebo, nocebo and context-related effects; including expectation, learning processes (classical conditioning and observational learning), reinforced expectations, mindset and personality traits. The neurophysiological mechanisms mainly include the endogenous opioid, the endocannabinoid and the dopaminergic systems. Neuroimaging studies have identified different brain regions involved such as the dorsolateral prefrontal cortex, the rostral anterior cingulate cortex, the periaqueductal gray and the dorsal horn of spine. From a clinical perspective, the manipulation of the healthcare context with the best evidence-based therapy represents an opportunity to trigger placebo effects and to avoid nocebo effects respecting the ethical code of conduct. From a managerial perspective, stakeholders, organizations and governments should encourage the assessment of the healthcare context aimed to improve the quality of physiotherapy services. From an educational perspective, placebo and nocebo effects are professional topics that should be integrated in the university program of health and medical professions. From a research perspective, the control of placebo, nocebo and context-related effects offers to the scientific community the chance to better measure the impact of physiotherapy on different outcomes and in different conditions through primary studies.

## Background

Placebo and nocebo effects embody complex and distinct psychoneurobiological phenomena where behavioural, neurophysiological, perceptive and cognitive changes occur during therapeutic encounter, between the (physio) therapist and the patient, in the healthcare context [1]. Specifically, placebos and nocebos can be defined as inert treatments or active treatments that are not therapeutically effective for the disease or condition under cure (active placebo or nocebo). These treatments, if administered in a therapeutic and healthcare context, can produce remarkable effects, known as “placebo effects” or “nocebo effects” [2]. “Placebo effects” are produced by a positive healthcare context that can ameliorate the patient’s symptoms [1, 3]. Conversely, “nocebo effects” are produced by a negative healthcare context that can elevate the patient’s symptoms [4–6]. These effects can also occur when an active and therapeutically effective treatment is administered: indeed, any healthcare treatment (active or inert) that is administered in any healthcare contexts (e.g., medical, rehabilitative) can trigger contextual-related effects. Placebo, nocebo and contextual-related effects have been used as models to examine the body-mind interaction by investigating the impact of these phenomena on different bodily processes, diseases and individual behaviour [7, 8].

Indeed, the healthcare context is not a vacuum, but it is an enriched relational space created by several elements [9–15] – defined as contextual factors – such as: (1) the physiotherapist’s professionalism, mindset and appearance; (2) the patient’s beliefs, experiences and expectation about the disease and the therapy; (3) the words, gestures and behaviour presented in the physiotherapist-patient relationship during the therapeutic encounter; (4) the rituality, the invasiveness and the overt application of the intervention; (5) the furniture, the architectural design and the overall impression of the clinic [16]. These contextual factors have been suggested as responsible for a large non-specific component of treatment efficacy, directly affecting the quality of patient’s health-related outcomes (e.g., pain, disability, satisfaction, and experience) [16–20]. For instance, the same treatment (e.g., exercise) if associated with physiotherapist’s positive verbal suggestion (e.g., “This exercise will ameliorate your symptoms”) can reduce the pain and increase the strength of a patient. Whereas, if associated with verbal suggestion of uncertainty (e.g., “This exercise sometimes might reduce symptoms”), could even worsen pain and strength [17, 21, 22].

**Fig. 1:**
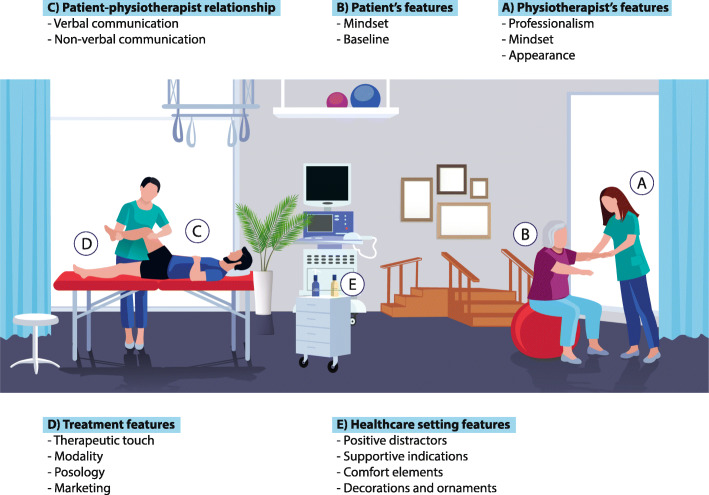
Contextual factors in clinical practice. The following contextual factors were accepted as effective modifiers of physiotherapy outcomes. For a review of this topic see reviews [16, 17, 19]. **a** Physiotherapist’s features: professionalism (expertise, qualification, reputation, education, trining); mindset (behaviour, beliefs, expectation, previous experience); appearance (attire, uniform, white coat, trustworthiness). **b** Patient’s features: mindset (expectation, previous experience, history of treatment, preference, desire, and emotion); baseline (level of symptoms, comorbidity, health condition, gender, age). **c** Patient-physiotherapist relationship: verbal communication (positive message, tone of voice, active listening, suggestions of support and encouragement, language reciprocity, warmth, attention, care, empathetic interaction); non-verbal communication (eye contact, facial caring expression, smiling, posture, gestures, head nodding, forward leaning, open body orientation). **d** Treatment features: therapeutic touch (emotional, empathetic, affective); modality (level of invasiveness, open/overt application, observational/social learning); posology (personalized treatment, treatment delivered by the same physiotherapist, cleanliness, adequate length of the consultation, punctuality, flexibility with patient’s appointments, timely and efficient treatment, adequate frequency, duration and follow-up of therapy); marketing (brand, prize, novelty, rituality). **e** Healthcare setting features: positive distractors (natural lighting, low noise levels, relaxing and soft music, pleasing aromas, adequate temperature); supportive indications (highly visible and easy to read signs, parking information, accessible entrances, clear and consistent verbal or written directions, information desks and accessible electronic information); comfort element (windows and skylights, private therapeutic settings, good access to services, convenient clinic hours, location, parking, and available and approachable support staff); decorations and ornaments (nature artworks, green vegetation, flowers, water, plants, garden, colour)

If compared with other healthcare professionals, the physiotherapist’s clinical action is deeply pervaded by placebo and nocebo effects for several reasons strongly related to the characteristics of physiotherapy administration [23]. Physiotherapists spend a significant amount of time with their patients in numerous sessions, intervening on different disorders (e.g., motor, cardio-respiratory, gastrointestinal, urogynecological or neurological), and therefore easily establishing an empathetic therapeutic relationship [24, 25]. Moreover, physiotherapists, more than medical doctors, interact with their patients using verbal (e.g., encouraging words) and non-verbal (e.g., communicative, non-therapeutic touch) elements [26, 27]. Every physiotherapy intervention, being it manual treatment, exercises or modalities, is naturally enriched by different contextual factors that can influence the trajectory of outcomes towards a positive or a negative result, depending on how they are managed by the physiotherapist [17] (Fig. 1).

Throughout the history of physiotherapy, placebo, nocebo and contextual-related effects have always been considered a challenging phenomena for two main reasons [28]. From a research perspective, these represented possible confounders capable of decreasing both internal and external validity of the studies conducted [29, 30]. From a clinical perspective, contextual factors symbolise troublesome and non-specific variables that can attenuate the therapeutic role of the specific interventions such as manual treatments, therapeutic exercise and modalities administration [31, 32].

It was at the end of the first decade of the twenty-first century that this attitude started to change. Indeed, the scientific community began to investigate the mechanisms of action of different therapeutic interventions like exercise and manual treatment of joint, soft and neural tissue, enlightening the weight of placebo and nocebo effects in such mechanisms and therefore their relevant role in physiotherapy [33, 34]. Emergent studies have suggested a mechanical and neurophysiological mechanism (peripheral, spinal and supraspinal) as the base of the effects induced by the therapeutic interventions adopted by the physiotherapists [35–37]. Considering the supraspinal mechanisms, placebo and nocebo effects were indicated as important top-down modulators of patient’s symptoms, mainly pain and motor performance [38–44], thus becoming elements that physiotherapists should attentively consider in their clinical practice [17].

Despite the growing interest in the physiotherapy field [16, 17], the knowledge and the awareness of using contextual factors to enhance placebo and avoid nocebo effects is still limited among physiotherapists [45]. The same unawareness has also been shown among other healthcare providers such as nurses and physicians [46, 47]. Moreover, the lack of a clear education about this professional topic in physiotherapy university courses reduces their perceived translational value and their relevance for physiotherapy practice [16, 48].

The aim of this narrative review is to offer a general overview of placebo, nocebo and contextual factors effects by: (1) presenting the psychological models behind their effects; (2) describing the neurophysiological mechanisms involved; (3) underlining the impact for the physiotherapy profession (clinical, managerial and educational); and (4) tracing lines for future researches.

To this end, the narrative review style has been selected, citing both primary studies (e.g., clinical trial) and secondary studies (e.g., systematic review, narrative review) on placebo and nocebo effects from different healthcare fields (e.g., medicine, nursing, physiotherapy) as previously reported [16, 17, 19]. Some studies specifically focus on the role of inert substances in producing positive (placebo) or negative (nocebo) effects, whereas others refer to the effect of the therapeutic context when an active treatment is administered (context-related effects).

## Main text

### The psychobiological determinants of placebo, nocebo and context-related effects

**Fig. 2:**
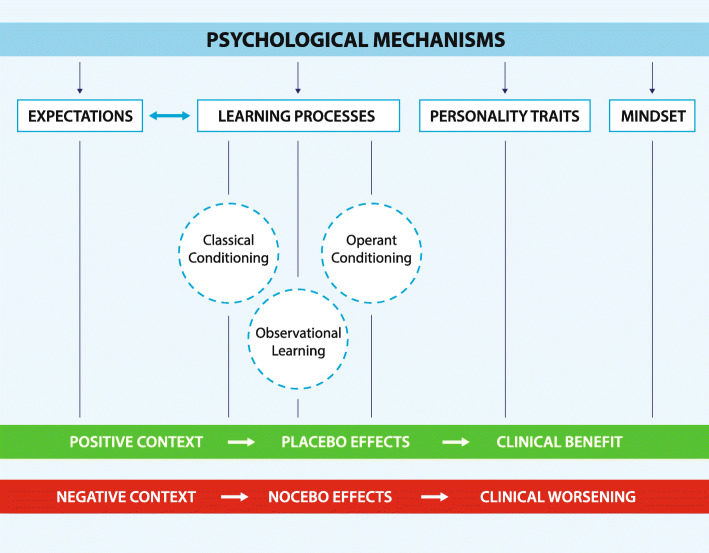
Psychobiological determinants of placebo, nocebo and context-related effects. Principal psychobiological determinants of placebo, nocebo and context related effects

There are several psychological mechanisms involved in placebo, nocebo and context-related effects including: expectation, learning processes such as classical conditioning and observational learning, reinforced expectations, mindset and personality traits (Fig. 2) [1].

Expectation refers to one’s anticipation of a future event. The expectations of individuals are a powerful modulator of their cognitive, emotional and physical experiences [49]. These are continuously shaped and updated according to the inputs coming from the surrounding environment [50]. Contextual factors summarized in Fig. 1 represent the key elements that are likely to influence patients’ expectations within the healthcare setting [17]. Verbal suggestions are the simplest and most direct way to shape expectations, therefore, a large body of evidence has used either positive or negative verbal information to modulate expectations and elicit placebo and nocebo responses. For example, Kam-Hansen et al. (2014) demonstrated that placebo labelled as active treatment (positive context expectation) and active treatment labelled as placebo (drug effect) had comparable healing effects on migraine attacks [51]. Similar findings were reported on post-surgical pain of thoracotomized patients [52] and on patients suffering from irritable bowel syndrome [53]. A recent study conducted on chronic neck pain patients has shown neck pain modulation according to contextual changes. Specifically, when positive verbal suggestions were associated with the neck manipulation, patients reported a significant reduction in pain. This was the case for both the real or the sham neck handling. A similar, but weaker effect was shown in relation to neutral information, whereas pain worsened when negative information was given [54].

Pavlovian classical conditioning [55] represents another mechanism that is strongly involved in placebo and nocebo effects, and associated positive and negative contexts [56–58]. Benedetti et al. (2003) showed that placebo administration (saline injection) after real drug preconditioning (sumatriptan injection) mimicked the effects of the drug on growth hormone (GH) secretion. In contrast, positive expectations alone without prior conditioning led to no effect on GH [59]. These findings, together with other classical conditioning studies [56, 60, 61], have demonstrated that verbally induced expectations alone have no effect on autonomous physiological processes such as hormonal plasma production and the immune system. However, a positive context created by a placebo administration after real drug preconditioning can elicit autonomic responses.

Conditioning can also function as an expectation booster, leading to reinforced expectations. To this end, a conditioning procedure can be used to create an association between an inert treatment and pain amelioration by reducing the intensity of the stimulation during the learning phase. During the recall phase, stimuli intensity is set back to baseline but is generally perceived as less painful [62]. Montgomery and Kirsch (1997) further investigated conditioning response when participants were overtly told the nature of the placebo and of the preconditioning. Interestingly, analgesic and hyperalgesic responses were no longer present. This suggests that conditioning alone was insufficient to elicit placebo and nocebo effects and that conscious expectations were necessary; indicating that conditioning served as a reinforcer to enhance expectation [63].

Observational learning represents another mechanism associated with placebo responsiveness. Colloca and Benedetti (2009) explored observational learning mechanisms in electric shock induced pain [64]. At first, participants observed a demonstrator taking part in the experiment and whom was instructed to shown analgesic and hyperalgesic effects when the painful stimuli were preceded by a green and by a red light, respectively. Then, participants underwent the same procedure they had observed and, as expected, they reported analgesia (pain decrease) and hyperalgesia (pain increase) in response to green and red cues accordingly. The same experiment was conducted using classical conditioning procedure as well as verbal suggestions alone. The magnitudes of placebo and nocebo effects were comparable between classical conditioning and observational learning procedures. On the contrary, the effect was much smaller for verbal suggestions alone. The role of observational learning in both positive social context (placebo) [65, 66] and negative social context (nocebo) [65–68] has been reiterated in later studies.

Up to date evidence has introduced a new mechanism involved in placebo and nocebo effects, namely operant conditioning [69]. This new paradigm consisted in rewarding and punishing participants when they responded ‘correctly’ to painful stimuli. Coloured cues preceded each stimulus, and dependently on the cue colour, the experimenter wanted the subjects to respond either with analgesia or hyperalgesia. Participants were rewarded or punished, via positive and negative writings displayed on the screen, accordingly on whether they responded as desired by the experimenter. When stimuli were presented in the absence of rewards and ‘punishments’, analgesia and hyperalgesia phenomena persisted, suggesting that operant conditioning functions as a learning process eliciting placebo and nocebo effects [69].

Other factors may interact with placebo and nocebo effects, including mindset and personality traits. Here, mindset refers to a broad set of viewpoints that compose one’s outlook on life. Multiple studies revealed that increasing the level of optimism in one’s mindset can positively change both subjective and objective measures of one’s health and wellbeing [70–72].

Concerning personality traits, suggestibility appears to make individuals more or less susceptible to the positive and/or negative context, leading to stronger placebo and nocebo responses [73]. Similarly, optimistic and pessimistic personality seems to facilitate placebo and nocebo effects, respectively [74]. Whereas, high trait and state anxiety tend to promote nocebo responses [75, 76]. With this knowledge in mind, no personality trait has yet been identified that can reliably predict if someone will respond to placebo (placebo responder) or not (placebo non responder) [1].

### The neurophysiological mechanisms of placebo, nocebo and context-related effects

**Fig. 3:**
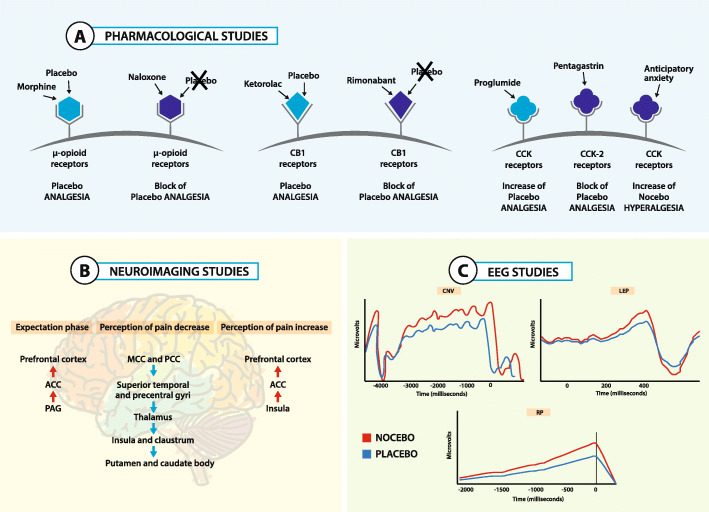
Neurophysiological mechanisms of placebo, nocebo and context-related effects. Principal neurobiological mechanisms of placebo, nocebo and context related effects. **a** Pharmacological studies. The opioid and cannabinoid systems are involved in placebo and nocebo effects. In some circumstances, placebo analgesia occurs through the activation of the opioid system and can be reversed by naloxone. In other circumstances, placebo analgesia occurs through the activation of the cannabinoid system and can be reversed by rimonabant. Anticipatory anxiety can activate the pro-nociceptive cholecystokinin (CCK) system, leading to nocebo hyperalgesia. The pro-nociceptive CCK effect can be reversed by proglumide and agonized by pentagastrin. **b** Neuroimaging studies. The activation and deactivation of different brain regions during placebo analgesia and nocebo hyperalgesia have been described trough different brain imaging studies. **c** Electrophysiological studies. Electroencephalographic (EEG) studies showed the dynamical studies showed the dynamical and temporal changes that occur in the brain before and after the administration of inert treatments

Over the past decade, the neurophysiological mechanisms underlying placebo, nocebo and context-related effects have begun to be extensively identified, using different approaches ranging from pharmacology to neuroimaging [17, 48, 77–79]. Pain and Parkinson Disease (PD) have been used as main models to understand the neurobiology of positive (placebo), negative (nocebo) and context-related effects [80, 81]. So far, three major questions have been addressed: (1) which are the neurobiological pathways activated by inert substances; (2) which are the brain regions that trigger these effects; and (3) which are the dynamical and temporal changes that occur in the brain before and after the administration of a placebo treatment (Fig. 3).

Pharmacological studies have demonstrated that the administration of an inert substance activates the endogenous opioid system and the endocannabinoid system. The observation that μ-opioid antagonists (e.g., naloxone) [56, 82, 83] and CB1-antagonist (e.g., rimonabant) [84] block some types of placebo analgesia has been documented by conditioning protocols using opioid drugs like morphine or cannabinoid drugs like ketorolac, respectively. Indirect confirmation of the involvement of the opioid system has been confirmed by the study of the anti-opioid action of cholecystokinin (CCK). Indeed, it has been demonstrated that CCK antagonist (e.g., proglumide) and CCK-2 agonist (e.g., pentagastrin) produce opposite effects on pain: the former enhances placebo analgesic effects while the latter disrupts them [85–89]. Also nocebo hyperalgesia seems to be modulated by the activation of the opioid system as it can be reversed by CCK antagonist. Moreover, placebos and nocebos also modulate the synthesis of prostaglandins, an important target of analgesic drugs [90]. On the whole, these pharmacological studies support the notion that inert substances and drugs may share common biochemical pathways.

Neuroimaging studies have identified different brain regions that contribute to placebo, nocebo and context related effects [91–99]. Different brain regions are activated during the “expectation phase”, when a positive or negative effect is foreseen, and during the “perception phase”, when analgesic or hyperalgesic effects are experienced. In particular, when a positive beneficial effect is *expected*, activation of anterior cingulate cortex (ACC), precentral and lateral prefrontal cortex and periaqueductal gray (PAG) has been documented. On the other hand, when placebo analgesia is *experienced* deactivation has been found in different brain regions such as the mid- and posterior cingulate cortex, superior temporal and precentral gyri, the anterior and posterior insula, the claustrum and putamen, and the thalamus and caudate body [100]. As for nocebo effects, when hyperalgesia is *expected and experienced*, an increased activity in different brain regions involved in pain processing and emotion regulation, such as prefrontal cortex (PFC), ACC and insula, has been documented [101–106]. Context-related effects have been extensively documented using the so-called “open-hidden” design, in which patients receive a real analgesic drug but they are either aware (open condition) or unaware (hidden condition) of receiving it. In these studies, it has been demonstrated that the open condition, that is a condition that maximizes the context-related effects, produced analgesic effect along with deactivation of the pain matrix and activation of dorsolateral prefrontal cortex (DLPFC) and rostral Anterior Cingulate Cortex (rACC). On the contrary, patients in the hidden condition, that is a condition that dramatically reduces the context-related effects, exhibited no changes in pain perception and no pain matrix deactivation [107].

Furthermore, the involvement of the dopaminergic system has been documented in pain [108, 109] and PD patients [110]. In placebo analgesia, an increase in dopamine binding to D2/D3 receptors and in opioid binding to μ-opioid receptors occurs in the nucleus accumbens, whereas a decreased binding to the same receptors is present in nocebo hyperalgesia [108, 109]. In PD, when patients experienced a motor improvement after a placebo administration, a large amount of dopamine was released in the dorsal motor striatum, suggesting a relationship between the amount of dorsal striatal dopamine release and clinical benefit. On the whole, these studies demonstrate that a complex network of brain regions is activated when placebos or nocebos are expected and positive or negative effects are experienced [111–113].

Recently, high temporal resolution techniques, such as electroencephalographic (EEG), has been used to determine changes in brain activity when placebo and nocebo phenomena arise. Studies on pain have revealed insights into the time-by-time and fast changes that occur before and after the administration of a sham treatment. Interestingly, as already documented by neuroimaging studies, placebos and nocebos change EEG brain activity during both the expectation and the perception phases. For example, the expectation of receiving no painful or painful stimuli respectively decrease or increase the amplitude of the contingent negative variation (CNV), an EEG slow negative wave that represents an objective measure of expectation of a specific incoming event (e.g., expectation of analgesia or hyperalgesia) [114]. Considering the “perception phase”, placebo treatments produce a decrease in laser-evoked potential (LEP), that represents an early measure of nociceptive processes, since it occurs after 200–250 ms after a painful stimulation [115]. Also motor electrophysiological potentials related to motor preparation and fatigue, such as the readiness potential (RP), can be affected by a placebo administration in healthy volunteers [116] and PD patients [117], demonstrating that the brain changes that occur when a placebo is administered can be triggered in different ways and in different times.

Even if modern brain imaging techniques have been fundamental in the understanding of the placebo and nocebo effects, further researches are needed to fully understand the underpinnings of these phenomena and the clinical implications and application of these findings.

### Clinical, managerial and educational implications for physiotherapy profession

In accordance with the evidence reported in this review, the analysis of placebo, nocebo and context-related effects may lead to new therapeutic strategies that are capable of improving the professional action of the physiotherapist and of influencing management and education.

From a *clinical perspective*, the integration of the contextual factors with the best evidence-based therapy represents an opportunity to stimulate placebo effects and to prevent nocebo effects; boosting therapy effectiveness. This would be done in accordance with the ethical and deontological code of conduct [118, 119]. Indeed, contextual factors pervade every clinical action (e.g., history taking, physical examination, therapy administration, prognosis communication and revaluation) and directly affect the quality of health-related outcomes [16, 17]. A positive context (e.g., an encouraging verbal suggestion during a joint manipulation [54], or an empathetic therapeutic relationship during a modalities administration [120]), can ameliorate patients’ clinical outcomes by triggering placebo effects. Instead a negative context (e.g., a detrimental verbal indication delivered during a joint manipulation [54] or during a muscle strength test [121, 122]), can worsen patients’ symptoms by stimulating nocebo effects. Examples of clinical application of contextual factors are presented in Table 1.

**Table 1:** Clinical application of contextual factors. The table presents examples of use of contextual factors aimed to enhance placebo effects and avoid nocebo effects. Adapted from [16, 17, 19]

Contextual factors	Actions to enhance (placebo effects)	Actions to avoid (nocebo effects)
(A) Physiotherapist’s features	• improve physiotherapist’s professionalism; • be aware of physiotherapist’s mindset; • promote physiotherapist’s appearance;	• overlook physiotherapist’s professionalism; • be unaware of physiotherapist’s mindset; • disregard physiotherapist’s appearance;
(B) Patient’s features	• examine patients’ mindset; • analyse patients’ baseline;	• neglect patients’ mindset; • ignore patients’ baseline;
(C) Patient-physiotherapist relationship	• manage verbal communication; • optimise non-verbal communication;	• neglect verbal communication; • ignore non-verbal communication;
(D) Treatment features	• amplify the rituality of treatments; • be aware of therapeutic touch; • consider the modality/posology of treatment; • use marketing of treatment;	• limit the rituality of treatments; • be unaware of therapeutic touch; • omit the modality/posology of treatment; • neglect marketing of treatment;
(E) Healthcare setting features	• adopt positive distractors and supportive indications; • use comfort elements, decorations and ornaments;	• avoid positive distractors and supportive indications; • omit comfort elements, decorations and ornaments;

From a clinical point of view, it is imperative to distinguish between the changes in patients’ symptoms resulting from placebo and nocebo effects, and those rising from other variables [123]. Possible confounders include the natural history (the spontaneous relief of the dysfunction and symptom modifications) and the regression to the mean (a statistical event caused by selection biases). Moreover, the patient’s and clinician’s confirmation biases during the description of clinical symptoms as well as unrevealed effects of simultaneous treatments have been reported as other confounders [1, 124].

From a *managerial perspective*, placebo, nocebo and context-related effects could help policy decision-makers during the design of the healthcare setting [16, 17]. Taking into account contextual factors offers the opportunity to significantly improve patients’ perception of physiotherapy services in terms of quality and overall satisfaction [16, 17]. Thus, at multiple levels (e.g., private or public services, inpatients or outpatients units), stakeholders, organizations and governments should encourage assessment and management of contextual factors [125, 126]. The positive context around the treatment (e.g., respect of timetable of physiotherapy treatment, a quiet setting) can impact the overall patients’ satisfaction and perception of their health care experiences by enhancing the attractiveness of a specific physiotherapy service [20, 127]. A positive healthcare context invites patients to choose, return to and recommend the physiotherapy service. Moreover, it increases the adherence to prescribed treatments and follow-ups [20, 127]. Instead, a negative healthcare context (e.g., a noisy environment, an overcrowded setting) improves the likelihood of patients’ dissatisfaction, the abandonment of the service and the withdrawing from the treatment plan [20, 127].

From an *educational perspective*, contextual factors (e.g., patient-clinician relationship) are underestimated during the majority of physiotherapy degrees. Awareness and practice of these professional topics should be strengthened and steadily integrated in teaching programs (e.g., core curriculum and core competence) and activities (e.g., skill-labs, role-playing), aiming to prepare the students for a better management of the psychosocial component in the clinical practice [16, 128]. In physiotherapy education, contextual factors help students to consider the therapeutic outcome as a complex, not predictable and nonlinear result of multiple interactions between different variables (e.g., clinicians, patients and healthcare setting) that evolve during the therapeutic encounter [129] in a positive or negative way through placebo and nocebo effects. Emerging evidence has suggested that awareness of contextual factors corresponds to good diagnostic skills and therapeutic reasoning [130–134], thus suggesting to academics and lecturers an additional teaching instrument for the development of students’ clinical ability.

### Emerging lines for future research

With an increased understanding of contextual factors, and placebo and nocebo effects, the physiotherapy scientific community can measure the impact of physiotherapeutic interventions with greater precision in primary studies.

In contrast to the simplicity of creating a pharmaceutical placebo (where the active component of the treatment is removed), devising physiotherapy placebos is a significant challenge. At first, the aim should be to identify the best physiotherapy placebos for existing treatments. Various studies tried to develop [135, 136] and validate [137–139] a sham placebo technique. The principle obstacles are the needs to assess the patients’ blinding, their expectations and their priori (real) inertness [140–142]. However, a novel sham procedure has recently been validated [143] and applied in clinical settings; both to patients with migraines [144] and those with cervicogenic headache [145], thus paving the way for a series of further studies.

Secondly, the impact of contextual factors on patient outcomes should be examined. The task of designing a proper trial continues to be inconclusive among scholars [78, 146]. Primary studies should follow a research agenda aimed to estimate the effect of contextual factors on various patient’s clinical outcomes and in various health conditions. Using randomized clinical trials, there is a need to compare the same physiotherapy treatment performed in a neutral and enriched context [16]; measuring the change of subjective (e.g., pain, disability, expectation and satisfaction) and objective (e.g., heart rate variability, salivary cortisol, electromyographic activity) outcomes.

Thirdly, researchers should examine the patient’s perception of contextual factors. Although the patient’s viewpoint on placebo interventions has been investigated through surveys and qualitative interviews [47, 147], only study in this field has accounted for the influence of the patient’s viewpoint so far; it investigated contextual factors in patients with musculoskeletal pain [148]. Moreover, an item bank of contextual factors has been presented to evaluate the patient’s viewpoint regarding the overall healthcare experience [149]. This preliminary finding represents an initial phase for the creation of a questionnaire that: classifies patients based on their preferred contextual factors; and helps clinicians to enrich the physiotherapy treatment with specific contextual elements.

## Conclusions

In summary (Table 2), this narrative review provides a stimulus for reflection on the role and strength of placebo, nocebo and context-related effects surrounding the administration of a physiotherapy treatment. On one hand, a positive healthcare context can significantly improve therapeutic effectiveness. On the other hand, a negative context can manifest adverse effects. However, the research on contextual factors is still at an early stage, and it constitutes an emerging field for investigation. Findings in this area of research could generate new psychologically appreciative treatments and thus create opportunity for growth for the physiotherapy profession.

**Table 2:** Key points on placebo, nocebo and context-related effects

**Take-home message**	
• Placebo and nocebo effects are psychoneurobiological phenomena respectively produced by a positive and a negative healthcare context around the treatment; • The healthcare context is composed by contextual factors such as the feature of: the physiotherapist; the patient; the patient-physiotherapist relationship; the treatment; and the healthcare setting. • The psychological determinants of placebo and nocebo effects include: expectation; learning (classical conditioning and observational learning); reinforced expectations; mindset; and personality traits. • The neurophysiological mechanisms of placebo and nocebo effects involve different systems (the endogenous opioid, the endocannabinoid, and the dopaminergic) and brain regions (dorsolateral prefrontal cortex, the rostral anterior cingulate cortex, the periaqueductal gray, and the dorsal horn of spine). • From a clinical perspective, the manipulation of the healthcare context with the best evidence-based therapy represents an opportunity to trigger placebo effects and to avoid nocebo effects respecting the ethical and deontological code of conduct. • From a managerial perspective, stakeholders, organizations and governments should encourage the assessment of the healthcare context aimed to improve the quality of physiotherapy services. • From an educational perspective, placebo and nocebo effects are professional topics that should be integrated in the university program of health and medical professions. • From a research perspective, the control of placebo, nocebo and context-related effects offers to the scientific community the chance to better measure the impact of physiotherapy on different outcomes and in different conditions through primary studies.	

## Data Availability

Not applicable.

## Abbreviations

GH: growth hormone
PD: Parkinson disease
CCK: Cholecystokinin
ACC: Anterior cingulate cortex
PAG: Periaqueductal gray
PFC: Prefrontal cortex
DLPFC: Dorsolateral prefrontal cortex
rACC: Rostral anterior cingulate cortex
EEG: Electroencephalographic
LEP: Laser-evoked potential
CNV: Contingent negative variation
RP: Readiness potential
